# Prognostic significance of interstitial fibrosis and tubular atrophy in biopsy-proven diabetic kidney disease: a single-center retrospective cohort study

**DOI:** 10.3389/fendo.2026.1854787

**Published:** 2026-07-08

**Authors:** Rui Zhang, Mingyan Wu, Hui Yang, Xingyan Zhou, Ziwei Guo, Haiyan Yu, Wenyu Song, Yi Bao, Yuxing Yang, Rui Yan

**Affiliations:** 1 Department of Nephrology, Affiliated Hospital of Guizhou Medical University, Guiyang, China; 2 Key Laboratory of Kidney Disease Pathogenesis Research and Transformation Application, Guizhou Medical University, Guiyang, China; 3 Department of Nephrology, The People’s Hospital of Qiandongnan Miao and Dong Autonomous Prefecture, Kaili, China; 4 Department of Rheumatology and Immunology, The People’s Hospital of Qiandongnan Miao and Dong Autonomous Prefecture, Kaili, China; 5 Department of Endocrinology, Affiliated Hospital of Guizhou Medical University, Guiyang, China

**Keywords:** biopsy, diabetic kidney disease, interstitial fibrosis and tubular atrophy, kidney outcomes, prognosis

## Abstract

**Background:**

Interstitial fibrosis and tubular atrophy (IFTA) is an important pathological feature of diabetic kidney disease (DKD). Its prognostic value in biopsy-proven DKD remains incompletely understood.

**Methods:**

In this retrospective study, 164 patients with type 2 diabetes and biopsy-confirmed pure DKD were followed for a median of 38 months. The primary composite kidney endpoint was initiation of kidney replacement therapy or kidney-related death. Predictors were selected using LASSO Cox regression. Random survival forest was used as an exploratory complementary analysis.

**Results:**

Thirty-four patients reached the composite kidney endpoint. After LASSO selection and multivariable adjustment, IFTA score 2/3 (vs. IFTA score 1, HR 3.96, 95%, CI 1.71–9.16, *P* = 0.001), 24-hour proteinuria (per 1 g/day increase; HR 1.52, 95%, CI 1.19–1.94, *P* < 0.001), and lower serum calcium (per 1 mmol/L increase; HR 0.140, 95% CI 0.041–0.481, *P* = 0.002) were independently associated with the composite kidney endpoint. In the random survival forest analysis, serum calcium, baseline eGFR, and IFTA were among the most important variables. Patients with IFTA 2/3 had significantly lower kidney survival than those with IFTA 1 (log-rank *P* = 0.0056).

**Conclusions:**

In biopsy-proven DKD, IFTA 2/3 was associated with the composite kidney endpoint. Higher 24-hour proteinuria and lower serum calcium were associated with poor kidney outcomes. Further studies are needed to confirm these findings and clarify their clinical implications.

## Introduction

Diabetic kidney disease is a common microvascular complication of diabetes and a leading cause of kidney failure worldwide. DKD is associated with substantial morbidity and mortality and places a major burden on healthcare systems. According to the International Diabetes Federation, approximately 589 million adults were living with diabetes worldwide in 2024, and this number is projected to increase to nearly 850 million by 2025 (1). As the prevalence of diabetes continues to rise, the number of patients with DKD is also expected to increase.

DKD is characterized by progressive kidney injury, including glomerular hyperfiltration, thickening of the glomerular basement membrane, mesangial expansion, and nodular glomerulosclerosis. However, several studies have shown that tubular injury may occur before the onset of microalbuminuria (2). Emerging evidence suggests that tubulointerstitial injury is closely associated with kidney disease progression and may have greater prognostic value than glomerular lesions (3, 4). The severity of tubulointerstitial damage can be assessed using the interstitial fibrosis and tubular atrophy (IFTA) scores (5, 6).

Several studies have reported an association between interstitial fibrosis and tubular atrophy and poor kidney outcomes in DKD. However, most studies were retrospective and included heterogeneous patient populations. The prognostic value of IFTA in biopsy-proven DKD remains incompletely defined. In addition, the relative importance of IFTA compared with traditional clinical and pathological factors remains unclear. Therefore, we conducted a retrospective cohort study of patients with biopsy-proven DKD. We evaluated the association between IFTA severity and kidney outcomes and explored the prognostic value of IFTA in this population.

## Method

### Study design and patients

This was a retrospective cohort study of patients with type 2 diabetes mellitus (T2DM) and biopsy-confirmed diabetic kidney disease at the Affiliated Hospital of Guizhou Medical University between June 2014 and July 2022. Inclusion criteria were: age >18 years, diagnosis of T2DM, and biopsy-proven DKD (no evidence of coexistent non-diabetic glomerular disease or other systemic renal diseases). T2DM was diagnosed according to the American Diabetes Association criteria or a documented history of T2DM. DKD was pathologically diagnosed and classified according to the Renal Pathology Society (RPS) 2010 classification system by at least two experienced renal pathologists. Exclusion criteria included: malignant tumors, coexistence with other primary or secondary glomerular diseases (e.g., IgA nephropathy, membranous nephropathy), active hepatitis, liver cirrhosis, hepatobiliary stones, estimated glomerular filtration rate (eGFR) <15 mL/min/1.73 m^2^ or ongoing dialysis at biopsy, urinary tumors, and incomplete clinical or follow-up data. The study was approved by the Ethics Committee of the Affiliated Hospital of Guizhou Medical University (No. 2025196K). As this was a retrospective study using anonymized data, the requirement for written informed consent was waived by the ethics committee. The study was conducted in accordance with the ethical principles of the Declaration of Helsinki and its later amendments.

### Clinical and laboratory data

Baseline clinical and laboratory data were collected at the time of kidney biopsy. Clinical variables included age, sex, duration of diabetes, smoking status, body mass index, blood pressure, and use of renin–angiotensin system inhibitors. Laboratory variables included serum creatinine, estimated glomerular filtration rate (eGFR), 24-hour proteinuria excretion, hemoglobin, serum albumin, serum calcium, serum uric acid, triglycerides, and total cholesterol. The eGFR was calculated using the Chronic Kidney Disease Epidemiology Collaboration (CKD-EPI) equation.

### Pathological evaluation

All kidney biopsies were examined by light microscopy, immunofluorescence microscopy, and electron microscopy. Pathological lesions were assessed according to the 2010 Renal Pathology Society classification of diabetic nephropathy, including glomerular class (I–IV), interstitial fibrosis and tubular atrophy (IFTA) score (0–3), interstitial inflammation score (0–2), and arteriolar hyalinosis score (0–2).

### Study outcome and follow-up

The primary outcome was a composite kidney endpoint consisting of initiation of kidney replacement therapy (dialysis or kidney transplantation) or kidney-related death. Patients were followed from the date of biopsy until the endpoint, loss to follow-up, or the end of the study period.

### Statistical analysis

Continuous variables were presented as mean ± standard deviation or median (interquartile range, IQR), depending on their distribution. Categorical variables were presented as numbers and percentages. Group differences in continuous variables were compared using one-way ANOVA or Kruskal–Wallis test. Categorical variables were compared with the chi-squared test or Fisher’s exact test. Patients with missing values were excluded from the analysis. A complete-case approach was used for all statistical analysis. LASSO Cox regression was applied to 36 baseline clinical, laboratory, and pathological variables. Predictor variables were standardized automatically before model fitting. The optimal lambda value (lambda.min) was selected using 10-fold cross-validation. Variables with non-zero coefficients were entered into the multivariable Cox proportional hazards model. The proportional hazards assumption was assessed using Schoenfeld residuals. No violation of the proportional hazards assumption was detected. Random survival forest analysis was performed as an exploratory complementary analysis to assess variable importance and potential non-linear associations. The model was constructed using 1000 trees with log-rank splitting. Kaplan–Meier survival curves were generated to compare kidney survival between groups. Differences were assessed using the log-rank test. All statistical analysis were performed using R software (version 4.2.1) and SPSS software (version 26.0; IBM Corp., Armonk, NY, USA). All tests were two-sided, and *P* < 0.05 was considered statistically significant.

### Patient and Public Involvement

Patients or the public were not involved in the design, conduct, reporting, or dissemination plans of our research.

## Result

### Baseline clinical and pathologic characteristics of the patients

A total of 164 patients with biopsy confirmed diabetic kidney disease were included in this study. Baseline demographic and clinical characteristics are presented in **Table 1**. The median age was 55 years (IQR 48–61 years), and 113 patients (68.9%) were male. The mean duration of diabetes was 108.9 ± 74.6 months. 57 patients (34.8%) had a history of smoking. The median eGFR was 45.9 mL/min/1.73 m^2^ (IQR 22.4–68.5 mL/min/1.73 m^2^), and the mean 24-hour urinary protein excretion was 4,647 ± 3,345 mg/24h. The median hemoglobin was 111 g/L (IQR 96–129 g/L), and the median serum albumin was 33.7 g/L (IQR 28.6–39.5 g/L). The mean serum calcium concentration was 2.12 ± 0.17 mmol/L. Renin–angiotensin system inhibitors were used by 109 patients (66.5%) (**Table 1**).

**Table 1:** Clinical features of the patients according to IFTA scores.

Variables	All (n=164)	IFTA1 (n=38)	IFTA2 (n=101)	IFTA3 (n=25)	*P* value
Age	55 (48-61)	53 (44-60)	55 (49-61)	57 (47-59)	0.393
Gender (male %)	113 (68.9)	26 (68.4)	68 (67.3)	19 (76.0)	0.702
Body mass index (kg/m^2^)	24.8 (22.57-26.82)	23.75 (21.98-27.16)	25.1 (22.90-26.92)	25 (22.40-26.60)	0.539
family history (n %)	49 (29.8)	12 (31.5)	27 (26.7)	10 (40)	0.416
Smoking (n %)	57 (34.8)	12 (31.5)	36 (35.6)	9 (36.0%)	0.895
SBP (mmHg)	149 (133-162)	138.5 (128.5-150)	150 (136-167)	149 (135.5-167)	0.006
DBP (mmHg)	86 (80-95)	84.5 (78.5-91.25)	86 (80-95)	89 (85.5-99)	0.148
Duration of diabetes (Months)	108.85 ± 74.57	88.18 ± 74.99	118.61 ± 73.56	100.80 ± 74.57	0.084
Fasting blood glucose (mmol/l)	9.57 (6.48-12.56)	9.89 (5.84-13.70)	9.87 (6.74-12.93)	9.38 (5.48-9.86)	0.213
Serum creatinine (µmol/l)	157.15 ± 73.27	123.52 ± 54.99	160.42 ± 69.47	195.07 ±91.43	0.000
e-GFR (ml/min/1.73m^2^)	45.87 (2.39-68.53)	68.44 (42.43-81.41)	43.85 (31.14-65.02)	38.77 (22.31-57.88)	0.000
Serum uric acid (µmol/l)	385.35 (321.75-445.20)	361.50 (321.00-440.50)	389.00 (325.05-450.95)	371.3 (316.30-462.13)	0.829
CysC	2.22 (1.67-2.67)	1.80 (1.41-2.23)	2.26 (1.78-2.66)	2.70 (1.84-3.18)	0.000
Serum albumin (g/l)	33.74 (28.57-39.47)	36 (28.71-39.72)	31.6 (28.10-38.45)	36.1 (30.55-45.40)	0.010
Triglyceride (mmol/l)	2.71 ± 1.68	2.94 ± 1.71	2.60 ± 1.61	2.81 ± 1.90	0.526
Total cholesterol (mmol/l)	5.06 (3.97-6.21)	4.565 (3.86-6.17)	4.97 (3.95-6.20)	6.01 (4.05-6.80)	0.292
LDL-c (mmol/L)	3.01 (2.26-4.17)	2.915 (2.182-4.007)	2.981 (2.230-4.170)	3.310 (2.665-4.595)	0.422
HDL-c (mmol/l)	1.10 (0.91-1.31)	1.129 (0.997-1.315)	1.090 (0.900-1.310)	1.160 (0.900-1.395)	0.587
24-hour proteinuria (mg/day)	4646.66 ± 3345.07	3676.77 ± 3010.11	4980.87 ± 3526.14	4770.67 ± 2771.22	0.120
Hemoglobin (g/l)	111 (96-129)	112.5 (102.5-131.25)	106 (91-127.5)	120 (105-130)	0.050
Calcium(mmol/l)	2.12 ± 0.17	2.17±0.16	2.08±0.17	2.2±0.16	0.000
sC3 (mg/l)	0.929 (0.813-1.070)	0.914 (0.918-1.057)	0.938 (0.817-1.065)	0.916 (0.759-1.080)	0.584
sC4 (mg/l)	0.295 ± 0.118	0.270 ± 0.072	0.299 ± 0.134	0.313 ± 0.101	0.307
sIgG (mg/l)	10.090 (7.321-11.457)	10.100 (7.315-11.500)	9.980 (7.310-11.300)	10.300 (8.110-12.550)	0.735
sIgA (mg/l)	1.940 (2.605-3.357)	2.705 (2.307-3.382)	2.430 (1.690-3.600)	2.650 (2.050-2.920)	0.675
sIgM (mg/l)	1.096 ± 0.595	1.119 ± 0.438	1.052 ± 0.610	1.239 ± 0.725	0.358
RAAS inhibitor(n %)	109 (66.5)	24 (63.2)	69 (68.3)	16 (64.0)	0.815
Kidney replacement therapy (n %)	28 (17.1)	1 (2.6)	24 (23.8)	3 (12.0)	0.012
Kidney-related death (n %)	6 (3.7)	1 (2.6)	3 (3.0)	2 (8.0)	0.480

SBP, systolic blood pressure; DBP, diastolic blood pressure; eGFR, estimated glomerular filtration rate; CysC, cystatin C; LDL-c, low density lipoprotein cholesterol; HDL-c, high density lipoprotein cholesterol; sC3, serum complement C3; sC4, serum complement C4; sIgG serum immunoglobulin G; sIgA, serum immunoglobulin A; sIgM, serum immunoglobulin M; RAAS, renin angiotensin aldosterone system.

According to the 2010 RPS classification, 38 patients (23.2%) had IFTA score 1, 101 patients (61.6%) had IFTA score 2, and 25 patients (15.2%) had IFTA score 3. Baseline demographic and clinical characteristics stratified by IFTA score are shown in **Table 1**.

Patients with IFTA 2 had lower hemoglobin and serum albumin levels. Patients with IFTA score 2 had lower serum albumin levels than those with IFTA score 1 (31.6 vs. 36.0 g/L, *P* = 0.010). Hemoglobin levels were also lower in the IFTA score 2 group (106.0 vs. 112.5 g/L, *P* = 0.050). No significant differences were observed among IFTA groups in age, sex, duration of diabetes, smoking status, body mass index, blood pressure, serum uric acid, triglycerides, total cholesterol, or 24-hour proteinuria. Patients with IFTA 2/3 had lower eGFR, higher serum creatinine, higher cystatin C levels, and greater 24-hour proteinuria than those with IFTA 1 (**Supplementary Table 1**).

### LASSO-selected predictors of the composite kidney endpoint

**Figure 1:**
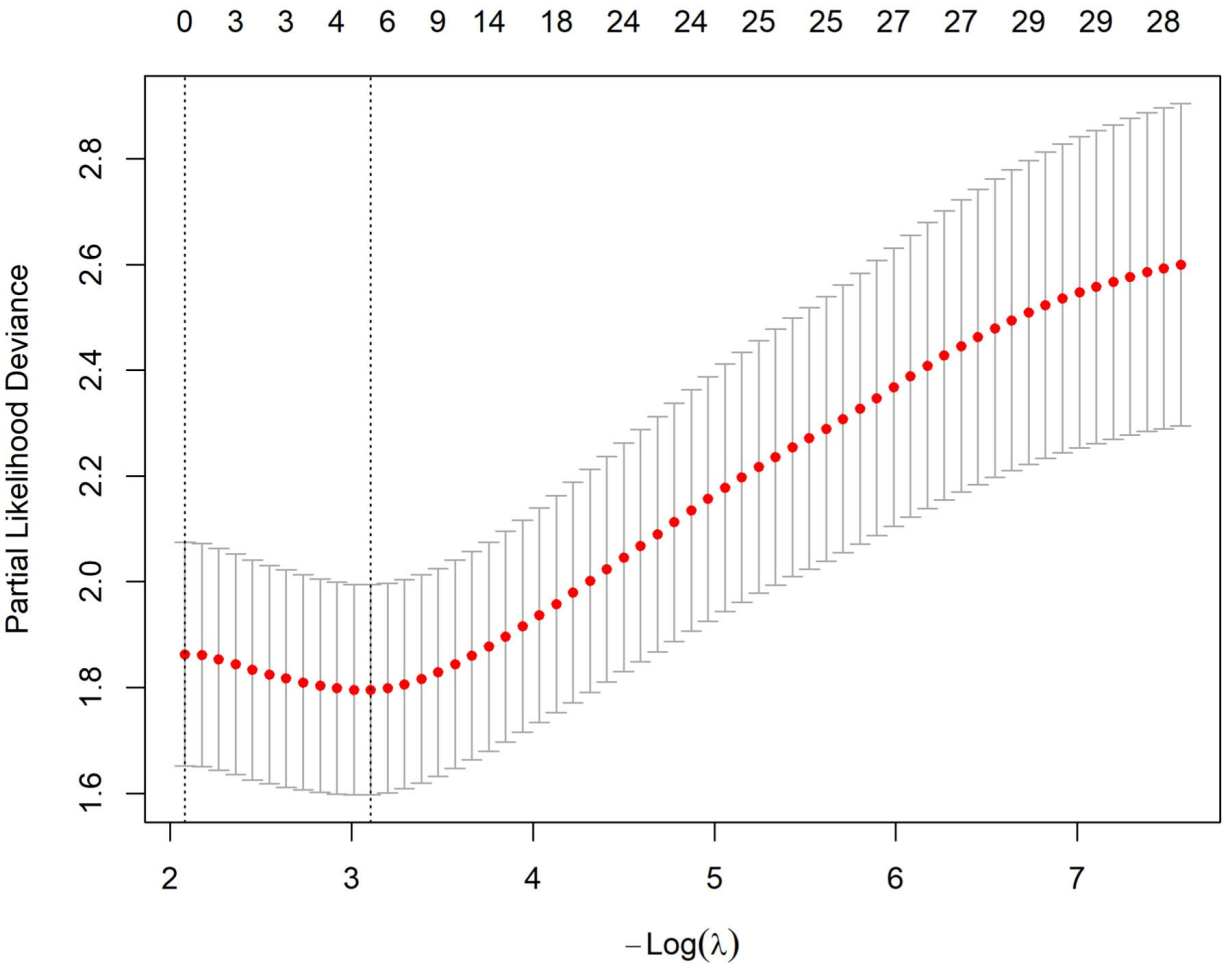
Variable selection using the LASSO Cox regression model.

LASSO Cox regression with 10-fold cross-validation was applied to 36 baseline clinical, laboratory, and pathological variables. Six variables with non-zero coefficients were selected: IFTA score, baseline eGFR, 24-hour proteinuria, serum calcium, serum IgA, and smoking status (**Figure 1**).

The six variables selected by LASSO were entered into a multivariable Cox proportional hazards model (**Table 2**). Because only 25 patients hadIFTA score 3,IFTA scores 2 and 3 were combined into a single group (IFTA 2/3) in the primary analysis. IFTA 2/3 (HR 3.96, 95% CI 1.71–9.16, *P* = 0.001), higher 24-hour proteinuria (HR 1.52, 95% CI 1.19–1.94, *P* < 0.001), and lower serum calcium (HR 0.140, 95% CI 0.041–0.481, *P* = 0.002) were independently associated with the composite kidney endpoint. Baseline eGFR, serum IgA, and smoking were not significantly associated with the endpoint.

**Table 2:** Multivariable Cox proportional hazards analysis of factors associated with the composite kidney endpoint.

Variables	HR (95% CI)	*P* value
IFTA 2/3 (ref: IFTA 1)	3.96 (1.71–9.16)	0.0013
24-hour proteinuria (per 1 g/24 h ↑)	1.52 (1.19–1.94)	<0.001
eGFR (per 1 mL/min/1.73 m^2^ ↑)	0.98 (0.97–1.00)	0.060
Serum calcium (per 1 mmol/L ↑)	0.14 (0.04–0.48)	0.002
Serum IgA (per 1 mg/L↑)	0.69 (0.41–1.17)	0.163
Smoking (ever vs. never)	1.63 (0.82–3.25)	0.163

The model included six LASSO-selected variables. Because of the limited number of patients with IFTA 3, IFTA 2 and 3 were combined into a single category (IFTA 2/3; reference: IFTA 1). The composite kidney endpoint was defined as initiation of kidney replacement therapy, or kidney-related death. HR, hazard ratio; CI, confidence interval; eGFR, estimated glomerular filtration rate; ↑, increase.

We performed a sensitivity analysis using the original three-level IFTA classification. Compared with IFTA 1, the adjusted HR was 4.58 (95% CI 1.05–20.01) for IFTA 2 and 2.08 (95% CI 0.39–11.18) for IFTA 3 (**Supplementary Table 2**). The confidence intervals were wide and overlapped substantially. No clear dose-response relationship was observed across IFTA scores.

### Exploratory complementary analysis using random survival forest

**Figure 2:**
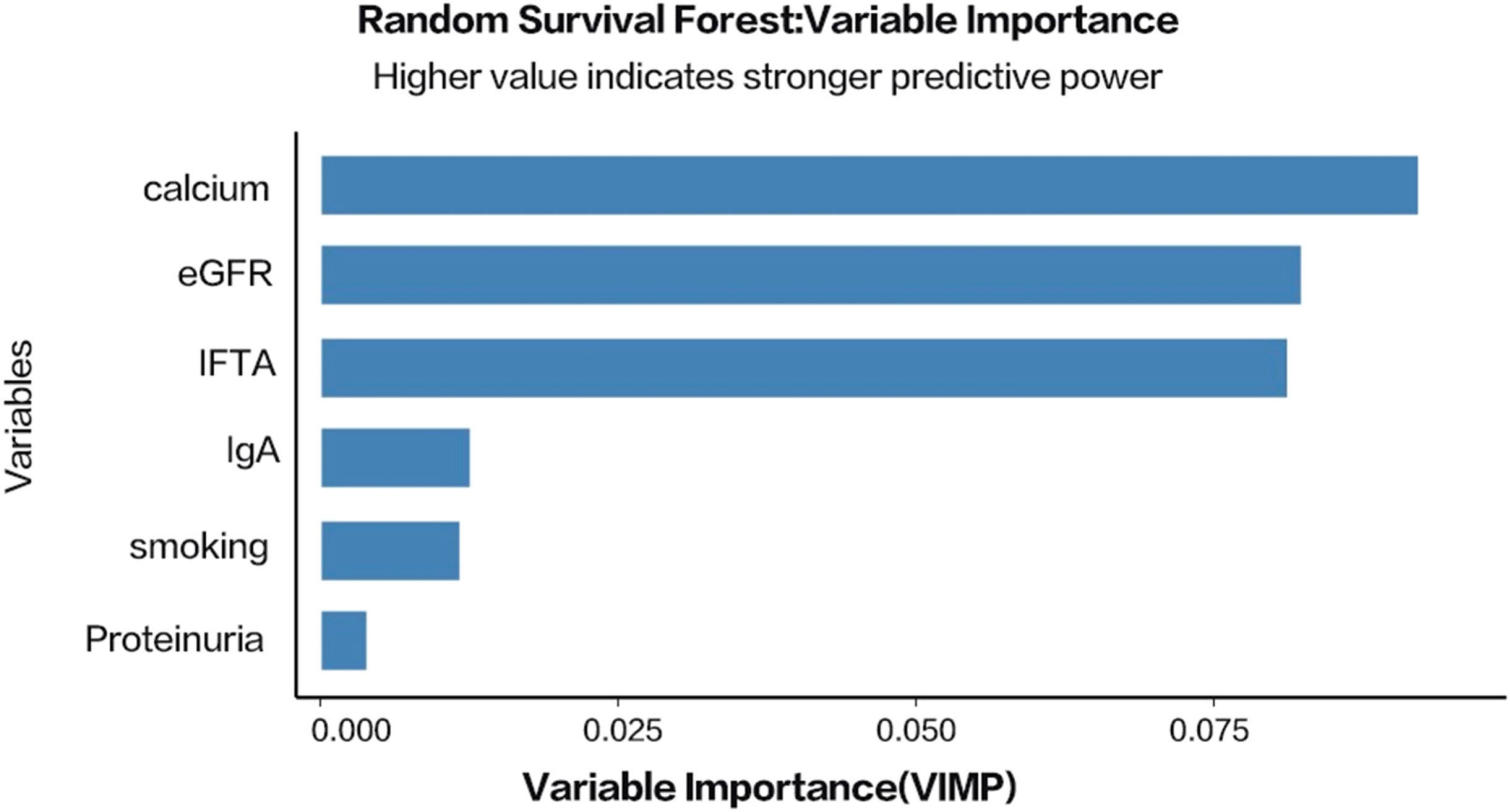
Random survival forest analysis. Variable importance (VIMP) ranking of the predictors included in the model.

We constructed a Random Survival Forest (RSF) model with 1000 trees. The terminal node size was set to 15, and the splitting rule was log-rank. Three variables were randomly selected as candidate splitting variables at each node. The model had an out-of-bag (OOB) C-index of 0.84 and a standardized OOB CRPS of 0.16. Permutation variable importance (VIMP) ranking is shown in **Figure 2**. The three variables with the highest variable importance were serum calcium, baseline eGFR, and IFTA score. Serum IgA, smoking status, and 24-hour proteinuria showed substantially lower importance values. These findings were consistent with the results of the multivariable Cox regression analysis.

### Non-linear associations with kidney disease progression

**Figure 3:**
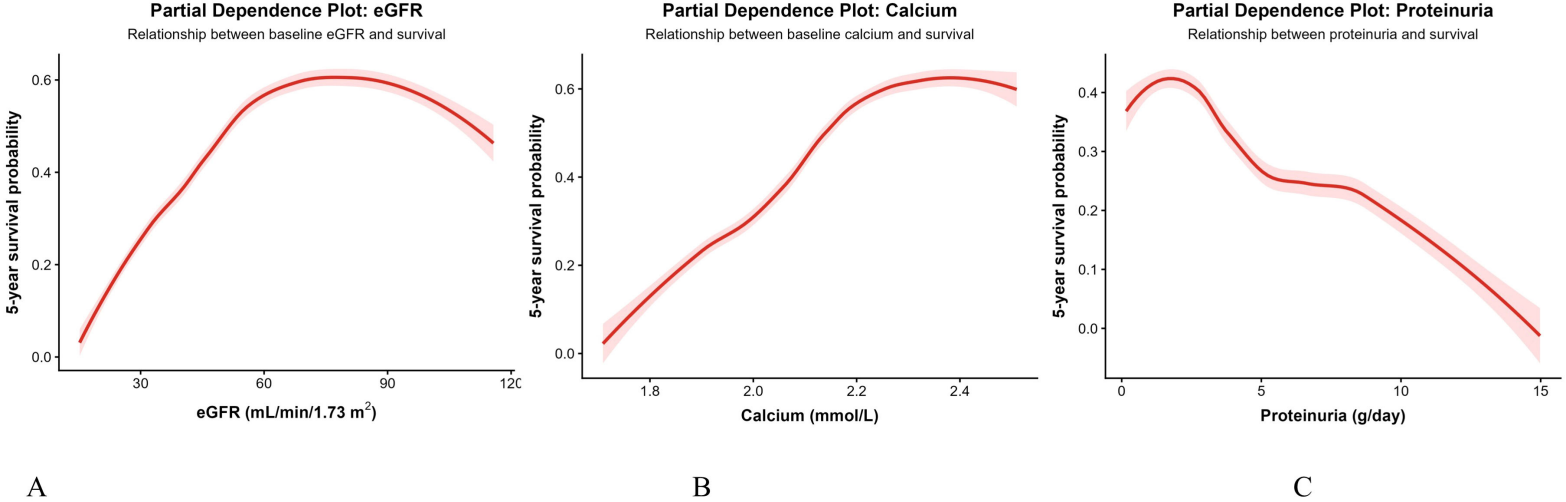
Partial dependence plots derived from the random survival forest model. The plots illustrate the estimated associations between selected variables and the composite kidney endpoint. **(A)** baseline eGFR, **(B)** Serum calcium; **(C)** 24-hour proteinuria.

Partial dependence plots derived from the random survival forest model were used as an exploratory analysis to examine potential non-linear associations between selected variables and the composite kidney endpoint (**Figure 3**). For baseline eGFR, the estimated risk decreased as eGFR increased to approximately 60 mL/min/1.73 m^2^ and then remained relatively stable. For serum calcium, the estimated risk decreased between 1.8 and 2.2 mmol/L and showed little change at higher levels. For 24-hour proteinuria, the estimated risk remained relatively stable at lower levels and increased with heavier proteinuria. These findings should be interpreted as exploratory because of the limited sample size and number of outcome events.

### Associations of IFTA with pathological characteristics and kidney survival

According to the RPS classification, 8 patients (4.9%) were classified as glomerular class IIa, 16 (9.8%) as class IIb, 124 (75.6%) as class III, and 16 (9.8%) as class IV. No patients were classified as class I. Interstitial inflammation score was 1 in 22 patients (13.4%) and 2 in 142 patients (86.6%). No patients had an interstitial inflammation score of 0. Arteriolar hyalinosis score was 0 in 34 patients (20.7%), 1 in 73 patients (44.5%), and 2 in 54 patients (33.5%) (**Table 3**).

**Table 3:** Pathological characteristics according to IFTA scores.

Variables	All (n=164)	IFTA1 (n=38)	IFTA2 (n=101)	IFTA3(n=25)	*P* value
Glomerular class(n (%))					0.000
IIa	8 (4.88)	4 (10.53)	2 (1.98)	2 (8.00)	
IIb	16 (9.76)	13 (34.21)	2 (1.98)	1 (4.00)	
III	124 (75.61)	21 (55.26)	86 (85.15)	17 (68.00)	
IV	16 (9.76)	0 (0.00)	11 (10.89)	5 (20.00)	
Interstitial inflammation (n (%))					0.001
1	22 (13.41)	12 (31.58)	8 (7.92)	2 (8.00)	
2	142 (86.59)	26 (68.42)	93 (92.08)	23 (92.00)	
Arteriolar hyalinosis(n (%))					
0	34 (20.73)	10 (26.32)	21 (20.79)	3 (12.00)	0.000
1	73 (44.51)	15 (39.47)	56 (55.45)	2 (8.00)	
2	57 (34.76)	13 (34.21)	24 (23.76)	20 (80.00)	

**Figure 4:**
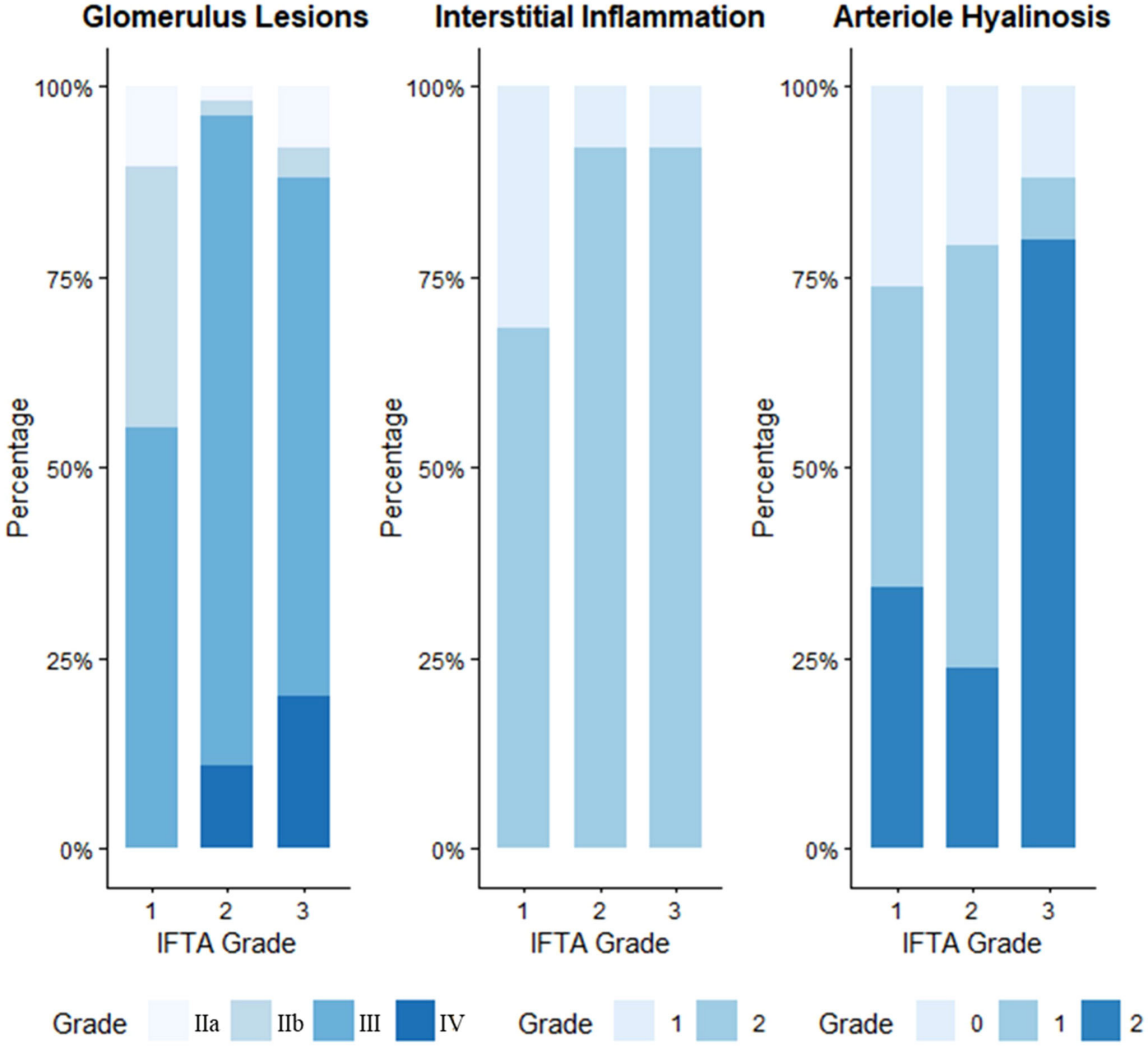
Distribution of RPS lesion scores stratified by IFTA scores with DKD patients. **(A)** Glomerular lesion class (IIa, IIb, III, IV). **(B)** Interstitial inflammation score (1, 2). **(C)** Arteriolar hyalinosis score (0, 1, 2).

Compared with patients with IFTA 1, patients with IFTA 2/3 had a higher frequency of interstitial inflammation score 2 and advanced glomerular lesions (class III or IV). All patients with IFTA 3 had interstitial inflammation score 2 and glomerular class III or IV (**Figure 4**).

**Figure 5:**
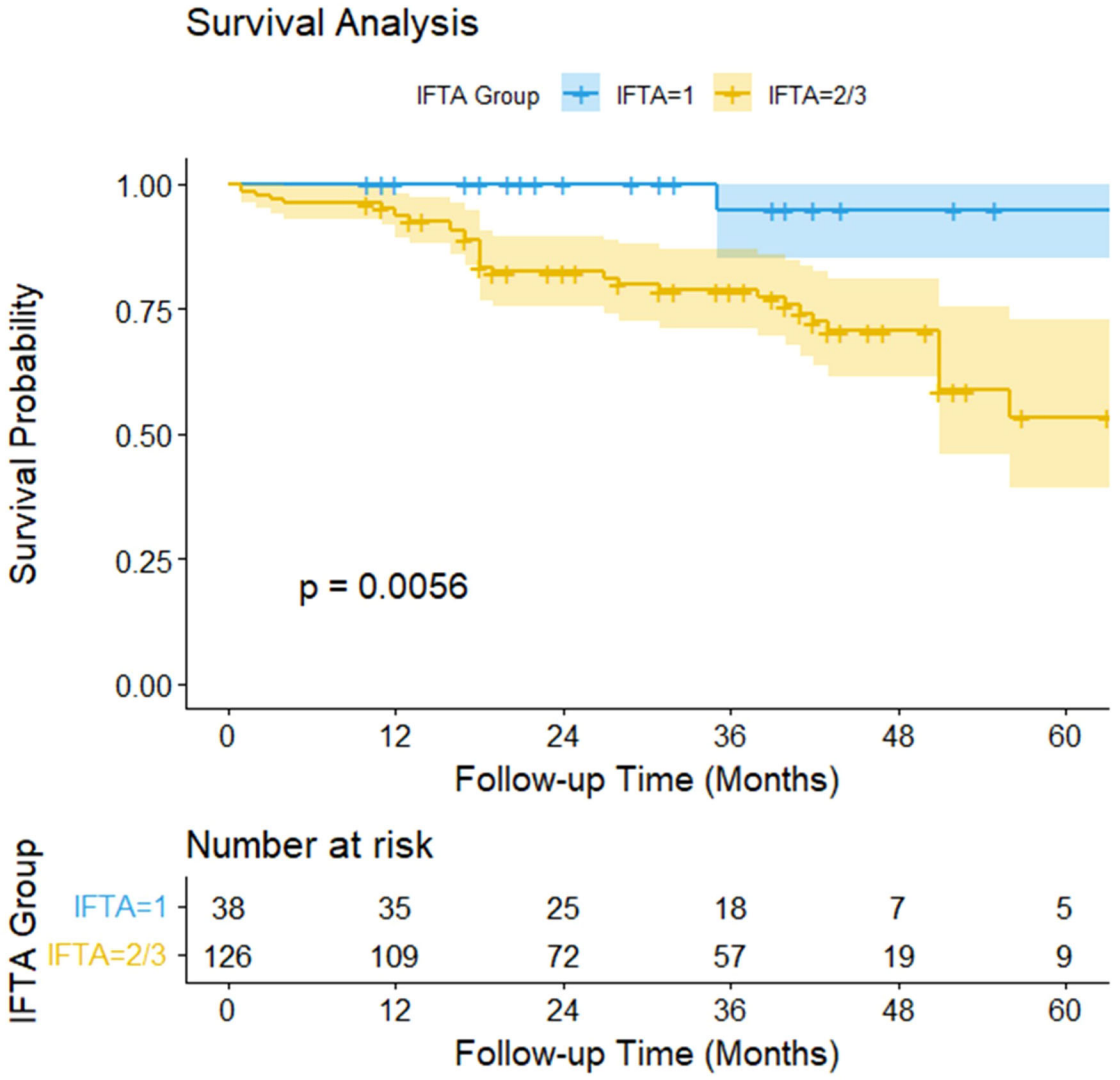
Kaplan–Meier curves of kidney survival according to IFTA scores (IFTA 1 vs. IFTA 2/3). Log-rank *P* = 0.006. Shading represents 95% confidence intervals.

Kaplan–Meier analysis showed a significant difference in kidney survival between the IFTA 1 and IFTA 2/3 groups (**Figure 5**). Patients with IFTA 2/3 were more likely to reach the composite kidney endpoint than those with IFTA 1 (log-rank *P* = 0.006).

## Discussion

In this retrospective cohort with biopsy-proven DKD, IFTA was strongly associated with kidney outcomes.it proved to be better than standard clinical factors and glomerular damage in various analysis. IFTA scores 2/3 were associated with an increased risk of the composite kidney endpoint. Higher 24-hour proteinuria and lower serum calcium were also independently associated with poor kidney outcomes. LASSO Cox regression identified these variables as important predictors, and similar findings were observed in the random survival forest analysis.

The role of tubulointerstitial pathology in DKD prognosis has received increasing attention over the past decade (7, 8), challenging the historical emphasis on glomerular lesions. Although the 2010 Renal Pathology Society classification focuses on glomerular lesion (9), accumulating evidence from large biopsy cohorts demonstrates that IFTA is associated with long-term kidney outcomes (10–13). A Japanese cohort of 233 patients with type 2 diabetes and biopsy-confirmed DKD showed that more severe IFTA independently predicted kidney events and overall mortality, the risk was higher in patients with low hemoglobin (14), it was consistent with our observation that patients with IFTA 2 had lower hemoglobin levels. In our study, IFTA 2/3 remained associated with composite kidney endpoint after adjustment for baseline eGFR, 24-hour proteinuria, and other covariates. Because no patients had IFTA score 0 and only 25 had IFTA 3, IFTA 2 and 3 were combined in the primary analysis. In a sensitivity analysis using the original three-level classification, the adjusted hazard ratios were 4.58 for IFTA 2 and 2.08 for IFTA 3, compared with IFTA 1. The confidence intervals overlapped substantially, and no clear dose-response relationship was observed. These findings support the use of the combined IFTA 2/3 group in the primary analysis. Kaplan–Meier analysis showed lower kidney survival in patients with IFTA 2/3 than in those with IFTA 1. In the random survival forest analysis, IFTA ranked among the variables with the highest variable importance, together with serum calcium and baseline eGFR. These findings further support the association between tubulointerstitial injury and kidney outcomes in biopsy-proven DKD.

In our cohort, patients with IFTA scores 2/3 more frequently exhibited glomerular class III and IV lesions. Similar findings were reported in a cross-sectional study from India, in which higher IFTA scores were associated with more advanced glomerular lesions, and higher IFTA scores were more common in patients with class III or IV DKD (10). Other studies have reported that tubulointerstitial lesions are closely associated with kidney outcomes in both diabetic and non-diabetic kidney diseases. In a biopsy-based study of DKD, Fukata et al. demonstrated that IFTA was associated with the risk of ESKD across different levels of proteinuria including both non-proteinuric and proteinuric patients (15). These findings support the prognostic importance of tubulointerstitial injury in DKD. The association between IFTA and kidney outcomes has also been reported in other kidney diseases. In studies of lupus nephritis, Rodelo et al. reported that IFTA and tubulointerstitial inflammation were associated with adverse kidney outcomes. Moderate-to-severe IFTA was associated with lower kidney survival (16). In kidney transplant recipients, Gabriel et al. reported that IFTA was associated with a decline in eGFR at 3 and 5 years post-transplant, the dynamic progression of IFTA served as an independent predictor of poor long-term graft outcomes (17). These findings suggest that IFTA is an important pathological feature associated with kidney outcomes across different kidney diseases. Assessment of IFTA may provide useful prognostic information in patients with DKD.

Our findings are consistent with the biological processes underlying tubulointerstitial injury in DKD. IFTA is associated with impaired tubular repair, chronic hypoxia, persistent inflammation, and fibrosis (18–21). Hyperglycemia, advanced glycation end-products, and activation of the renin–angiotensin system have been linked to tubulointerstitial fibrosis through myofibroblast activation and extracellular matrix accumulation (5, 22–24). Consistent with these mechanisms, we observed that IFTA 2/3 were associated with more severe interstitial inflammation and more advanced glomerular lesions, suggesting that IFTA reflects the overall severity of kidney injury in DKD. Pathological changes of IFTA may develop early in the disease course and may contribute to progressive loss of kidney function (22, 25). Together, these observations support the prognostic value of IFTA in patients with DKD.

The 24-hour proteinuria was independently associated with kidney endpoint, in keeping with its role as an indicator of podocyte injury and glomerular barrier dysfunction (26, 27). However, its lower ranking in the random survival forest analysis suggests that tubulointerstitial injury and other systemic factors may also contribute to kidney outcomes. As proposed by Letizia et al. (20), tubulointerstitial lesions may play an increasingly important role in kidney function decline during the progression of DKD.

Lower serum calcium was independently associated with composite kidney endpoint in our cohort. This association remained after adjustment for albumin. However, serum calcium can be affected by many factors, including mineral metabolism, nutritional status, and medication use. In a large cohort of 15,755 adults with CKD stages 3–5, Cynthia et al. reported that lower baseline serum calcium levels were associated with faster decline in kidney function (28). However, two cross-sectional studies by Yan and Wang reported a positive association between serum calcium levels and the presence of DKD (29, 30). In our analysis, serum calcium was independently associated with the composite kidney endpoint. Partial dependence plots suggested that the association between serum calcium and kidney outcomes might not be linear. However, this observation should be interpreted with caution because serum calcium may be influenced by several clinical factors that were not fully captured in this study. Further studies are needed to clarify the relationship between serum calcium and kidney outcomes in DKD.

Smoking and serum IgA were retained in the LASSO model but were not independently associated with the composite kidney endpoint in the multivariable Cox analysis. Smoking has been linked to kidney injury through oxidative stress and hemodynamic changes in previous studies (31–33). However, the prognostic significance of smoking and serum IgA was not confirmed in our cohort. Further studies are needed to clarify their roles in DKD progression.

Our study has several limitations. First, this was a single-center retrospective study with a limited sample size and relatively short follow-up period. Second, this was a biopsy-based cohort, and the included patients represented a selected subgroup of DKD, these findings may not be generalizable to all patients with type 2 diabetes and presumed DKD who do not undergo kidney biopsy. Third, Only a small number of patients received SGLT2 inhibitors or DPP-4 inhibitors. No patients received GLP-1 receptor agonists or mineralocorticoid receptor antagonists. Therefore, these treatments were not included in the analysis.

In biopsy-proven DKD, IFTA 2/3 was independently associated with the composite kidney endpoint. Higher 24-hour urinary protein excretion and lower serum calcium were also associated with poor kidney outcomes. These findings support an association between tubulointerstitial injury and kidney outcomes in DKD. Further studies are needed to confirm these findings and clarify their clinical implications.

## Data Availability

The original contributions presented in the study are included in the article/**Supplementary Material**. Further inquiries can be directed to the corresponding author.
