# Supplementary material for: Prognostic significance of interstitial fibrosis and tubular atrophy in biopsy-proven diabetic kidney disease: a single-center retrospective cohort study
Source: Front Endocrinol (Lausanne). 2026 Jul 8;17:1854787. doi: 10.3389/fendo.2026.1854787 (PMC13388118; doi:10.3389/fendo.2026.1854787)
Supplement: Supplementary file 1 [file DataSheet1.pdf]

Supplementary

	IFTA 1 (n = 38)	IFTA 2/3 (n = 126)	P -value
Age	53.00 (44, 60)	55.00 (49, 61)	0.433
Gender (male %)	26 (68.4)	87 (69.0)	1.000
Body mass index (kg/m²)	23.75 (21.98-27.16)	25.05 (22.83, 26.70)	0.263
family history (n %)	12 (31.5)	37 (29.4)	0.953
Smoking (n %)	12 (31.5)	45 (35.7)	0.783
SBP (mmHg)	138.50 (128, 150)	150.00 (136, 166)	0.002
DBP (mmHg)	84.5 (78.5-91.25)	87.50 (80.00, 95.75)	0.116
Duration of diabetes (Months)	88.18 ± 74.99	115.08 ± 73.61	0.051
Fasting blood glucose (mmol/l)	9.89 (5.88, 13.32)	9.57 (6.62, 12.40)	0.766
Serum creatinine (μmol/l)	123.52 ± 54.99	167.29 ± 75.22	0.001
e-GFR (ml/min/1.73m²)	68.44 (42.43-81.41)	43.58 (30.69, 62.57)	<0.001
Serum uric acid (μmol/l)	361.50 (321.00-440.50)	388.50 (322.00, 449.62)	0.601
CysC	1.80 (1.41-2.23)	2.31 (1.78, 2.84)	<0.001
Serum albumin (g/l)	36 (28.71-39.72)	33.15 (28.50, 39.23)	0.297
Triglyceride (mmol/l)	2.94 ± 1.71	2.64 ± 1.67	0.328
Total cholesterol (mmol/l)	4.565 (3.86-6.17)	5.08 (3.98, 6.23)	0.529
LDL-c (mmol/L)	2.915 (2.182-4.007)	3.05 (2.27, 4.17)	0.478
HDL-c (mmol/l)	1.129 (0.997-1.315)	1.10 (0.90, 1.31)	0.529
Urinary protein excretion (mg/day)	3676.77 ± 3010.11	4939.16 ± 3396.60	0.041
Hemoglobin (g/l)	112.5 (102.5-131.25)	109.00 (94.25, 128.00)	0.293
Calcium(mmol/l)	2.17±0.16	2.10 ± 0.17	0.039
sC3 (mg/l)	0.914 (0.918-1.057)	0.93 (0.81, 1.07)	0.726
sC4 (mg/l)	0.270 ± 0.072	0.30 ± 0.13	0.150
sIgG (mg/l)	10.100 (7.315-11.500)	10.04 (7.41, 11.40)	0.827
sIgA (mg/l)	2.705 (2.307-3.382)	2.58 (1.81, 3.33)	0.257
sIgM (mg/l)	1.119 ± 0.438	1.09 ± 0.64	0.784
RAAS inhibitor(n %)	24 (63.2)	85 (67.5)	0.767

Supplementary Table S1. Baseline characteristics according to IFTA group.

	Adjusted HR	95% CI
IFTA 1	1.00	Reference
IFTA 2	4.58	1.05–20.01
IFTA 3	2.08	0.39–11.18

Supplementary Table S2. Sensitivity analysis using the original three-level IFTA classification.
Adjusted for eGFR, serum calcium, IgA, proteinuria, and smoking. IFTA grade 1 was used as the reference group.

	χ^2	df	P value
IFTA	3.25	2	0.20
eGFR	1.32	1	0.25
Serum calcium	3.06	1	0.08
Serum IgA	0.20	1	0.65
24-hour proteinuria	0.07	1	0.80
Smoking	0.03	1	0.87
Global test	10.57	7	0.16

Supplementary Table S3. Test of the proportional hazards assumption for the multivariable Cox model

	IFTA 1 (n=38)	IFTA 2 (n=101)	IFTA 3 (n=25)	Total (n=164)	P value
Kidney-related death, n (%)	1 (2.6)	3 (3.0)	2 (8.0)	6 (3.7)	0.480
Kidney replacement therapy, n (%)	1 (2.6)	24 (23.8)	3 (12.0)	28 (17.1)	0.012
Loss to follow-up, n (%)	10 (26.3)	22 (21.8)	4 (16.0)	36 (22.0)	0.670

Supplementary Table S4. Distribution of Clinical Outcomes According to IFTA scores.
